# Uncommon osseous involvement in multisystemic sarcoidosis

**DOI:** 10.4103/0256-4947.57175

**Published:** 2009

**Authors:** Mohammad R. Rajebi, Armin Shahrokni, Marya Chaisson

**Affiliations:** a From the University of Tennessee Medical Center-Nuclear Medicine, Derby, Connecticut, United States of America; b From the Griffin Hospital-Medicine, Derby, Connecticut, United States of America

A 50-year-old man with a past medical history of nephrolithiasis presented with right upper quadrant and flank pain. The physical examination was unremarkable. Routine lab investigations were normal. Angiotensin-converting enzyme levels were elevated, but hypercalcemia was not detected. A chest radiograph showed multiple bilateral nodules associated with hilar fullness and widening.

**Figure 1:**
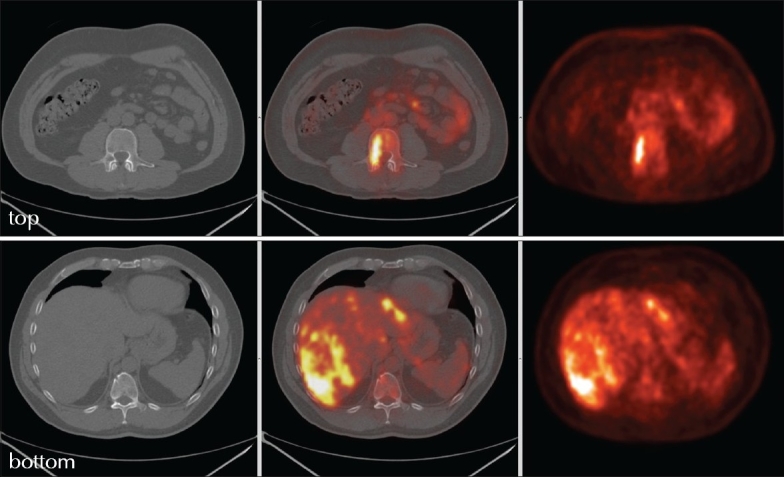
Lumbar vertebral (top) and hepatic involvement (bottom).

**Figure 2:**
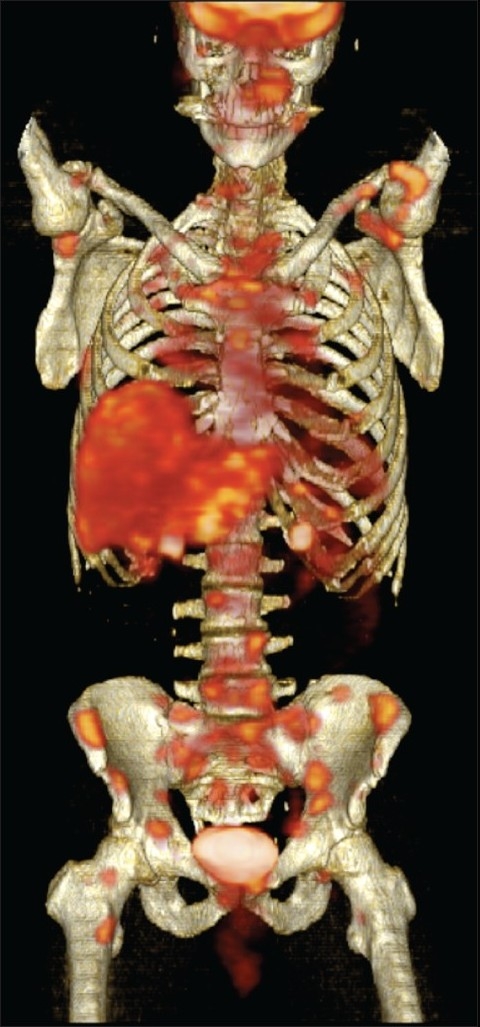
Maximum intensity projection image showing multiple foci abnormal hypermetabolic activity throughout the axial and appendicular skeleton.

**Figure 3:**
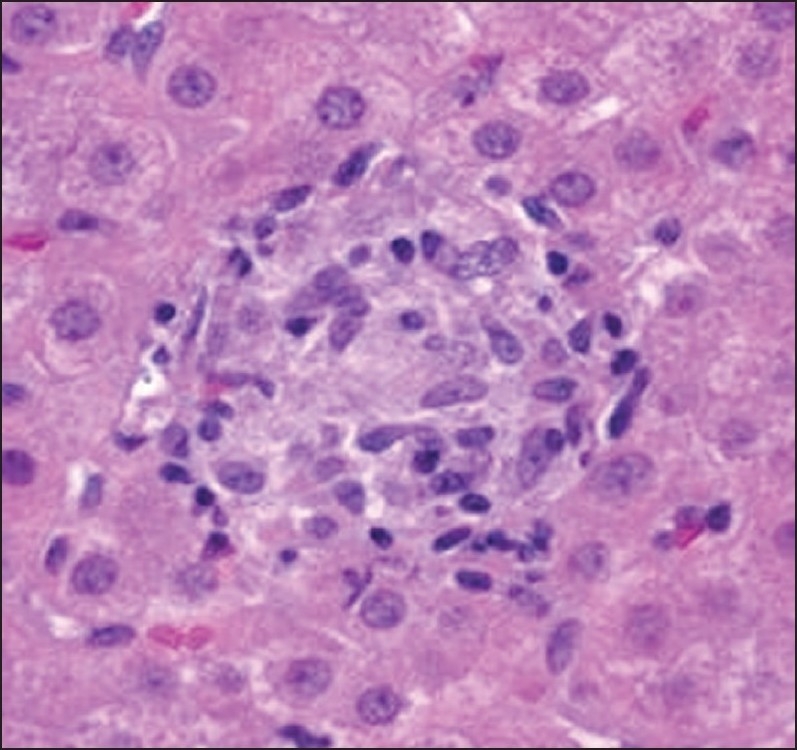
Hepatic biopsy showing non-caseous granuloma.

A CT scan of the chest and abdomen showed mediastinal and hilar lymphadenopathy, pulmonary nodules, and multiple ill-defined low-density lesions in the liver and spleen. A whole body fluorodeoxyglucose positron emission tomography/computed tomography (FDG PET/CT) showed multiple FDG-avid foci throughout the vertebrae, left maxilla, right infraorbital region, left sphenoid, the scapulae, left clavicle, sternum, ribs, bilateral iliac crest, and proximal femora in addition to FDG-avidity in the pulmonary nodules and hilar area (Figures 1 and 2). Biopsy of the right iliac crest and a percutaneous liver biopsy revealed non-caseating granulomatous inflammation with asteroid bodies. The biopsy was negative for acid-fast bacilli, fungi, and malignancy (Figure 3).

Sarcoidosis is a multi-systemic disease of unknown etiology that predominantly affects the lungs and intrathoracic lymph nodes. Non-caseating granulomas are the pathological hallmark of sarcoidosis and disorders in T-cell function play a role in the pathogenesis of this disease.^1^

Osseous sarcoidosis has been reported in 1% to 14% of patients with sarcoidosis and usually involves the small bones of the hands and feet.^2^ Involvement of vertebrae and long bones is extremely uncommon. With nonspecific accumulation of FDG into inflammatory cells, the disease can be visualized. The degree of FDG uptake has been related to activity of disease.^3^

The percentage of patients with osseous sarcoidosis may be underestimated. The FDG PET/CT scan may be a sensitive tool for evaluating the extent of bone involvement. Corticosteroids remain the major therapeutic option for symptomatic musculoskeletal sarcoidosis, but randomized, controlled trials are lacking.^4^
